# Recent Advances in Electrochemical Immunosensors with Nanomaterial Assistance for Signal Amplification

**DOI:** 10.3390/bios13010125

**Published:** 2023-01-11

**Authors:** Avinash V. Police Patil, Yu-Sheng Chuang, Chenzhong Li, Ching-Chou Wu

**Affiliations:** 1 Department of Bio-Industrial Mechatronics Engineering, National Chung Hsing University, No. 145, Xingda Rd., South Dist., Taichung City 402, Taiwan; 2 Department of Biochemistry and Molecular Biology, Tulane University, 1324 Tulane Ave., New Orleans, LA 70112, USA; 3 Innovation and Development Center of Sustainable Agriculture, National Chung Hsing University, No. 145, Xingda Rd., South Dist., Taichung City 402, Taiwan

**Keywords:** affinity reaction, immunosensors, nanomaterials, electrochemistry

## Abstract

Electrochemical immunosensors have attracted immense attention due to the ease of mass electrode production and the high compatibility of the miniature electric reader, which is beneficial for developing point-of-care diagnostic devices. Electrochemical immunosensors can be divided into label-free and label-based sensing strategies equipped with potentiometric, amperometric, voltammetric, or impedimetric detectors. Emerging nanomaterials are frequently used on electrochemical immunosensors as a highly rough and conductive interface of the electrodes or on nanocarriers of immobilizing capture antibodies, electroactive mediators, or catalyzers. Adopting nanomaterials can increase immunosensor characteristics with lower detection limits and better sensitivity. Recent research has shown innovative immobilization procedures of nanomaterials which meet the requirements of different electrochemical immunosensors. This review discusses the past five years of advances in nanomaterials (metal nanoparticles, metal nanostructures, carbon nanotubes, and graphene) integrated into the electrochemical immunosensor. Furthermore, the new tendency and endeavors of nanomaterial-based electrochemical immunosensors are discussed.

## 1. Introduction

Biosensors, consisting of a biomolecular recognition part and a transducer part, can selectively and sensitively quantify the concentration of a target analyte in complicated samples. The recognition part can be classified into catalytic elements, such as enzymes and whole cells, and affinity elements, such as antibodies, recombinant proteins, and synthetic recognition molecules, including peptides, oligonucleotides, peptide nucleic acids, aptamers, G-quadruplexes, molecularly imprinted polymers, etc. [1]. Immunosensors using antibodies as biorecognition elements have become the most widely used analytical device and testing strip for the requirement of clinic diagnostics, drug residue, microbial contamination in foods, and environmental toxin monitoring. Unlike the request of substrate addition for enzymatic catalysis, the antibody–antigen immunoreaction can directly produce the effect of physical signals (charges, weight, impedance, or optical absorbance) on a transducer. Moreover, the immunosensors generally have a superior specific reaction and a lower dissociation constant to analytes than aptasensors. Electrochemical transducers have attracted huge attention in constructing immunosensors due to the advantages of inexpensive cost, good compatibility with miniature and portable electrical readers, and ease of large-scale electrode production. Electrochemical immunosensors provide good selectivity and sensitivity when using a variety of signal probes, including enzymes, redox mediators, and nanomaterials, for amplifying immunoreaction results. Multiple electrochemical methodologies, including potentiometry, cyclic voltammetry (CV), differential pulse voltammetry (DPV), square wave voltammetry (SWV), anodic stripping voltammetry (ASV), and electrochemical impedance spectroscopy (EIS), are frequently used to quantify the immunoreaction results. Many reviewing works have elucidated the advanced development of electrochemical immunosensors in the detection issues of tumor markers [2], cancer biomarkers [3,4,5], cytokine tumor necrosis factor [6], microbial pathogens (bacteria and viruses) [7,8,9], anti-inflammatory drugs [10], pesticides and herbicides [11], antibiotics [12], and aflatoxin [13]. These biomarkers, inflammation factors, drug residues, and pathogens are required to be detected at an ultralow concentration (<ng/mL) in practical use, implying requirement of ultrasensitive immunosensors. Therefore, improving the sensing properties and lowering the detection limit becomes essential for immunosensor construction. 

**Scheme 1:**
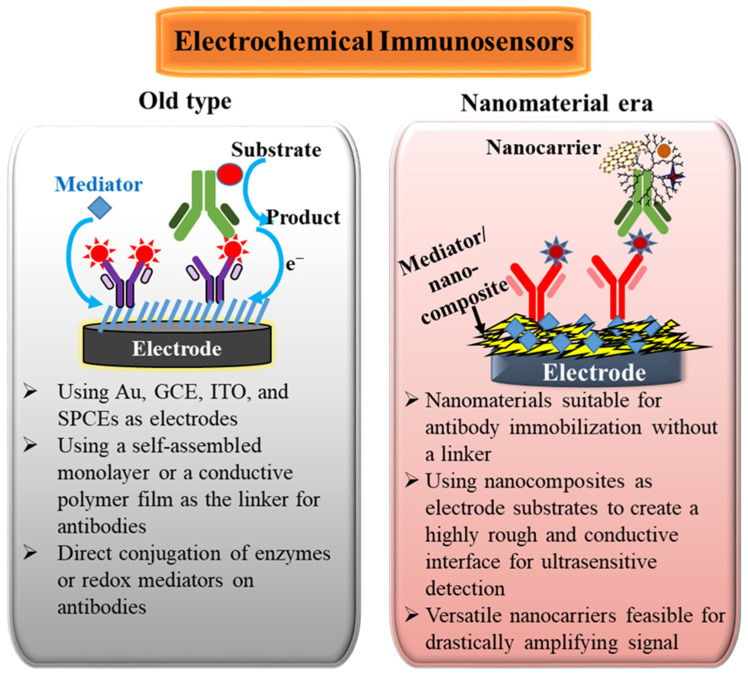
Comparisons and features before and after merging nanomaterials in electrochemical immunosensors.

In the past twenty years, nanoscale materials have had exponential growth. The nanomaterial adoption has also affected the development of electrochemical immunosensors. Scheme 1 shows the comparisons and features before and after merging nanomaterials in electrochemical immunosensors. The goals of applying nanomaterials in electrochemical immunosensors can be considered from two aspects. One is the promotion of the electron-transfer rate, which can increase the electrocatalytic ability of electrodes for electroactive probes and reduce the impedance of the electrode/electrolyte interface to amplify the redox current. The other is to supply a high surface-to-volume ratio, which can increase the number of biorecognition molecules immobilized on electrode surfaces and the number of labels adsorbing on nanomaterials, resulting in the amplification of immunological interaction. Generally, the nanomaterial-modified electrodes and nanomaterial-conjugated labels can improve the sensing characteristics to obtain more sensitive immunosensors.

The operational strategies of electrochemical immunosensors can be divided into label-free and label types. In a label-free format, the analyte (antigen) concentration is directly quantified after forming antigen-antibody complexes, which can hinder the electron-transfer rate and diffusive flux of mediators in the electrode/electrolyte interface to obtain a decreasing current detected by CV, DPV, or SWV and an increasing impedance measured by EIS. Therefore, label-free immunosensors are vital to require high conductivity and large surface-to-volume ratio nanomaterials to provide a higher initial current for subsequent current decrement. In contrast, label-type immunosensors, such as sandwich immunoreaction, adopt nanomaterial-labeled detection antibody (DAb) and capture antibody (CAb) immobilized on an electrode surface to increase the selectivity and sensitivity. The nanomaterial-conjugated DAb can bind to the antigen caught by the CAb of the electrode surface. The DAb-conjugated nanomaterials can act like a nanocarrier, which may have modification of the electroactive mediators and catalyzers, to promote the redox efficiency of mediators or the catalytical capacity of substrates for electrochemical response amplification.

**Figure 1:**
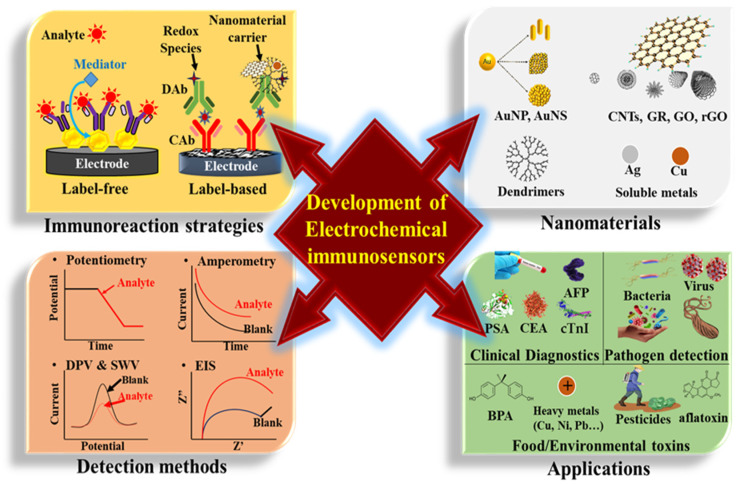
The concerned issues in nanomaterial-based electrochemical immunosensors.

This review summarizes a variety of nanomaterial-based electrochemical immunosensors reported in recent articles over the last five years [14,15,16,17]. Importantly, we discuss the effect and usage of nanomaterials (such as gold nanoparticles (AuNPs), gold nanostructures (AuNSs), carbon nanotubes (CNTs), graphene (GR), graphene oxide (GO), reduced graphene oxide (RGO), dendrimers, quantum dot (QD), silver nanoparticles (AgNPs), and their nanocomposites) on electrochemical immunosensors. As shown in Figure 1, the scheme shows the significant issues of fabricating an ultrasensitive electrochemical immunosensor, including the types of used nanomaterials, operational strategies of immunoassay, adequate electrochemical methods, and sensor applications. 

## 2. Immunoreacting Strategies and Electrochemical Detection

In addition to adopting nanomaterials to promote the sensing results, the adequate combination of immunoreacting strategies and electrochemical detection techniques can obtain more straightforward immunoreacting procedures, shorter response time, fewer reagents, more significant signals, and superior selectivity. The detecting techniques can be classified as label-free and label-based methods. The label-free detection is suitable for directly quantifying the results of one-step antigen-CAb immunoreaction. The required electrochemical techniques have potentiometric, DPV, SWV, and EIS, frequently reported in previous studies. The label-based methods are suitable for the sandwich and competitive immunoreaction by using the catalyzer-labeled DAb or DAb-conjugated nanomaterials to report the antigen-CAb immunoreaction. Amperometry, DPV, SWV, and ASV are the main electrochemical techniques for this method. 

### 2.1. Label-Free Electrochemical Immunosensor

In label-free immunoassay, the electrochemical detector can directly quantify the bound antigen number by measuring the change on the surface potential, redox current of mediators, or the electron-transfer rate of mediators after immunoreacting concentration-varied antigens. Different electrochemical or electric detectors can sense the change in the electrochemical properties of the electrode/electrolyte interface. 

#### 2.1.1. Potentiometry-Based Immunosensors

The bound antigens may cause a potential shift of the electrode surface, increase the thickness of the biorecognition layer, and produce steric hindrance to ionic flux from the bulk electrolyte to the ion-selective membrane. Potentiometry and field-effect transistor (FET) techniques are sensitive to the potential change of the electrode surface. The potential shift results from the charged functionality accumulation, such as NH^2+^, NH^3+^, COO^−^, PO_4_^3−^, of analytes on the electrode/electrolyte interface or the blocking effect of antigen–antibody immunoreaction on the ionic flux. The electronic detecting mechanism and electrode fabrication of immune FET devices can be referred to in these review works [18,19,20]. In principle, silicon-based nanowires, CNT, 2D nanomaterials (GR, MoS_2_), and conductive polymer can be used as the gate electrode for antibody immobilization. After immunoreaction, the drain current is related to the change in the gate potential. It is worth noting that the extended gate organic electrochemical transistors, using a conductive polymer as the hole or electron channel, have great potential for developing flexible and wearable biosensors [21].

**Figure 2:**
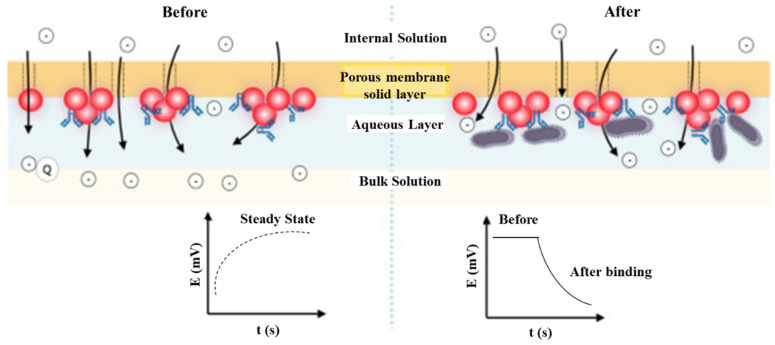
The scheme shows the effect of surface blocking on the ion flux by potentiometric detection in the immunoreacting interface. Redrawn from [23]. © Elsevier publishing.

In contrast, potentiometry presents an intuitive detection to monitor the equilibrium potential of the working electrode versus a reference electrode with a high input impedance voltammeter and a concentration difference between the inner and outer of the ion-selective membrane. Silva et al. [22] fabricated a disposable paper-based potentiometric immunosensor for label-free detection of *Salmonella typhimurium.* The potential shift of poly(3,4-ethylene dioxythiophene): polystyrene sulfonate (PEDOT: PSS)-coated paper is derived from the blocking effect of the ionic flux caused by *Salmonella*-antibody conjugation. Similarly, Silva deposited AuNPs on an ion-selective membrane for anti-*Salmonella* immobilization. The potential shift was attributed to the blocking effect of the antigen-antibody conjugation in the ionic flux [23]. Generally, the real-time potential drift can be instantly measured by potentiometry, as shown in Figure 2.

#### 2.1.2. Voltammetry-Based Immunosensors 

**Figure 3:**
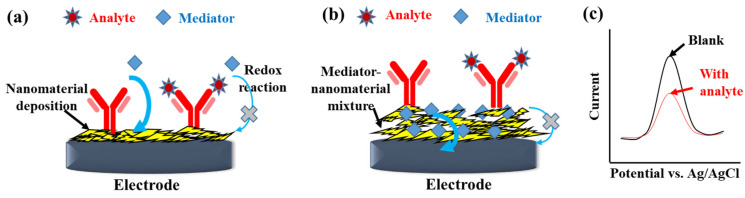
Scheme of detection mechanism of the label-free electrochemical immunosensors with mediators spiked in the electrolyte (**a**) or the modification layer (**b**). (**c**) The illustration shows the decrease of peak current with the analyte immunoreaction when performing DPV or SWV measurement.

Voltametric techniques, including CV, normal pulse voltammetry, DPV, SWV, AC voltammetry, and ASV, control the potential between a working electrode and a reference electrode to measure the redox current. Among these techniques, CV, DPV, and SWV are frequently applied in electrochemical sensors [24] and label-free affinity biosensors. The addition of mediators, such as negatively charged [Fe(CN)_6_]^3−/4−^ [25] or positively charged [Ru(NH_3_)_6_]^3+^ [26] in the electrolyte, are vital for label-free detection [27], as shown in Figure 3a. Furthermore, the mediators, such as methylene blue [28], Prussian blue [29,30], thionine (Thi) [31,32], or ferrocene [33], can also be co-immobilized in the nanomaterial-based modification layer of electrodes to probe the change in the interfacial impedance of the electrodes (Figure 3b). For example, Farzin et al. [32] grafted Thi and anti-prostate-specific antigen (PSA) CAb on the histamine-GO/multiwalled CNT (MWCNT)/glassy carbon electrode (GCE) as a mediator to probe the immunoreaction of prostate-specific antigen (PSA) directly. The reductive peak current of Thi measured by DPV was inversely proportional to the PSA concentration. The electroactive mediators immobilized on an electrode surface could not only supply a native redox signal but also amplify the redox current of [Fe(CN)_6_]^3−/4−^. Dong et al. [29] electrodeposited Prussian blue and the anti-organophosphorus pesticides CAb-adsorbed AuNPs on screen-printed carbon electrodes (SPCEs) to increase the surface conductivity of the immunosensor, which is beneficial for obtaining a sensitive DPV peak current of [Fe(CN)_6_]^3−/4−^. Furthermore, different mediators can be co-immobilized on electrode surfaces to produce a synergistic effect. Zhao et al. [30] deposited AuNPs–Prussian blue composites and Thi on a GCE to construct a sensitive capsaicinoids immunosensor with a limit of detection (LOD) of 0.01 ng/mL. The Thi/Prussian blue–AuNPs/GCE could produce a larger DPV peak current of [Fe(CN)_6_]^3−/4−^ than the Prussian blue–AuNPs/GCE and the TH/GCE. 

The antigen–antibody conjugation could increase steric hindrance and electrostatic influence of the biorecognition layer, which decreases the electron-transfer rate and diffusive flux of the mediators. The mediator response to the interfacial change of affinity biosensors can be measured by voltammetry. However, the CV signal involves capacitive and Faradaic currents, which lower the signal-to-noise ratio. In contrast, DPV can reduce the effect of non-Faradaic current and diffusion-controlled behavior on the measured current to obtain a peaked shape output, as shown in Figure 3c. In a reversible system, the peak potential of DPV is close to the formal potential. After the affinity reaction, the peak current height decreased with increasing concentration of the nonconductive antigen. Lan et al. [25] placed platinum nanoparticle (PtNP)-decorated RGO@polystrene nanospheres on a glassy carbon electrode (GCE) for anti-carcinoembryonic antigen (CEA) immobilization. DPV was used for the label-free detection with an inverse correlation between the peak current of [Fe(CN)_6_]^3−/4−^ and the CEA concentration. Verma et al. [34] developed a label-free immunosensor for detecting the oral cancer biomarker IL8 by utilizing AuNP-RGO-modified indium tin oxide (ITO) electrodes. CV and EIS were used to realize the effect of nanomaterial modification on the electrochemical properties of electrodes during preparation. DPV was used to obtain the relation between the peak current of [Fe(CN)_6_]^3−/4−^ and the IL8 concentration. Presently, DPV is the most prevailing voltammetry for the detection of electrochemical affinity biosensors.

SWV is reverse pulse voltammetry, which obtains the current difference between the forward and reverse sampling current during the staircase potential shift. For a reversible reaction, SWV is more sensitive than DPV because the reverse pulses near the formal potential can produce a reducer to increase the anodic current, which enlarges the current difference. SWV has a similar waveform to that of DPV (as shown in Figure 3c). Moreover, SWV is more compatible with mediator-immobilized detection (Figure 3b) than mediator-suspended detection (Figure 3a) in label-free immunosensors because the antibody immobilization and the surface blocking would increase the interfacial impedance. The phenomenon may cause a considerable over-potential to the redox reaction of suspension-type mediators, reducing the contribution of reverse current to the current difference of SWV. Zheng and Ma immobilized methylene blue on an alginate calcification layer and then deposited AuNPs-RGO, peptide, and albumin-Pd-polydopamine (PDA) nanocomposite for the label-free detection of metalloproteinase-7. The SWV peak current of methylene blue was sensitive to the modification procedures and metalloproteinase-7 concentrations [35]. Moreover, Li et al. [5] reviewed the development of electroactive species-based immunosensors, which is interesting for further study. 

#### 2.1.3. EIS-Based Immunosensors

**Figure 4:**
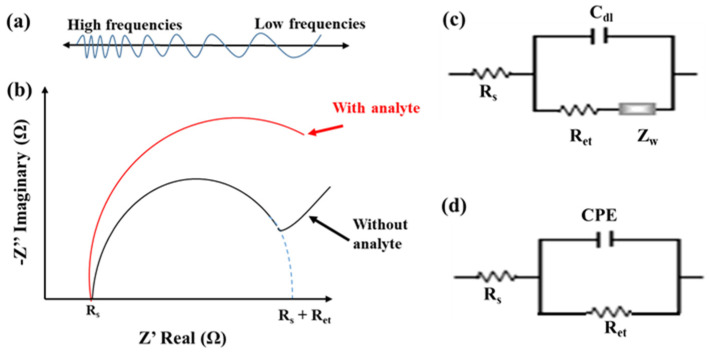
(**a**) The DC equilibrium potential superimposed with a frequency-varied AC voltage for EIS measurement. (**b**) Typical Nyquist plots measured by EIS with and without analyte immunoreaction. (**c**) The Randels equivalent circuit. (**d**) 1R//C equivalent circuit.

EIS is another prevalent electrochemical technique for label-free detection of affinity-based biosensors. It can examine sensitively the slight change in the electrochemical properties of the electrode/electrolyte interface, including the electron-transfer kinetics and the diffusive flux. The EIS operation is to superimpose a frequency-varied AC voltage, typically 5 or 10 mV, on the equilibrium potential of a redox couple, commonly using equimolar [Fe(CN)_6_]^3−/4−^, as shown in Figure 4a. The EIS data can be plotted in a Bode plot, the magnitude (|Z|) and phase (θ) of impedance versus frequencies, or a Nyquist plot, the imaginary part (Z”) to the real part (Z’) of the impedance [36]. Figure 4b, (the without-analyte curve), shows a typical Nyquist plot obtained from EIS measurement at a bare electrode or a modification-loose immunosensor. The semicircle and linear regions of the Nyquist plot are associated with the electron-transfer kinetics measured at high frequencies and the diffusion behavior obtained at low frequencies, respectively. The semicircle radius approximates half of the electron-transfer resistance (R_et_). The Randles equivalent circuit, consisting of four elements—the solution resistance (R_s_), the diffusion-related Warburg impedance (Z_w_), the pure capacitance of the electrical double layer (C_dl_), and R_et_, shown in Figure 4c—is used to explain the electrochemical properties of electrode/electrolyte interface.

Furthermore, after reacting analytes on a dense modification immunosensor, the steric hindrance and the electrostatic repulsion of the modification layer to the negatively charged mediator, [Fe(CN)_6_]^3−/4−^, can cause a high impedance with a slow electron-transfer rate and little diffusive behavior. The Nyquist plot only presents a semicircle (the with-analyte curve of Figure 4b). The Z_w_ can be eliminated from the Randles circuit. Moreover, due to the complex biorecognition layer, the constant phase element (CPE) was used to replace the C_dl_ to elucidate the inhomogeneity of the electrode surface. The impedance of the CPE can be presented by Z_CPE_(ω) = Z_0_ (jω)^−α^, where Z_0_ is a constant, j is an imaginary number, ω is the angular frequency, and 0 < α < 1. When α is closer to 1, the CPE becomes more capacitive. A simplified parallel equivalent circuit with R_et_ and CPE, called 1R//C, as shown in Figure 4d, is used to represent the interfacial impedance. Empirically, the change in R_et_ value is more sensitive than the CPE value in the Faradic impedance measurement.

Several strategies, such as nanocomposite deposition [37], the linker layer of low surface impedance [38], antibody-oriented immobilization [39,40], and convective transportation of analytes [41], have been reported to increase the sensing properties of EIS-based immunosensors. The nano-structured or nanomaterial-deposited surface can increase the electrode area to immobilize more antibodies. Ganganboina et al. [37] cast GR quantum dots@Au-polyaniline nanowires on a Pt electrode for impedimetric detection of CEA with a limit of detection (LOD) of 0.01 ng/mL. The types and deposition methods of nanomaterials used for electrochemical immunosensors are explored in Section 3 in detail, Nanomaterials for Electrode Modification.

### 2.2. Label-Based Electrochemical Immunosensor

**Figure 5:**
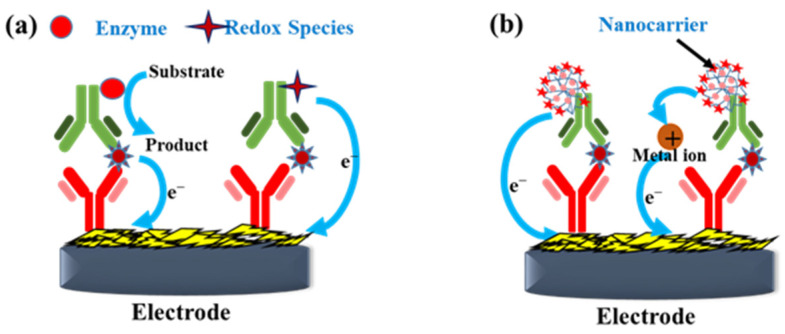
Schemes of the enzyme or redox species-labeled DAb (**a**) and the nanocomposite-based carrier for DAb immobilization (**b**) used in sandwich immunosensors.

Label-based immunosensors can be achieved by sandwich immunoreaction or competitive immunoreaction. The enzyme- or redox species-labeled DAbs report the antigen–CAb conjugation. Horseradish peroxidase (HRP) [42], glucose oxidase (GOD) [43,44], or alkaline phosphatase (ALP) [45], are conjugated to DAb to catalyze the corresponding substrates, and then the products are frequently detected by amperometry or voltammetry, as shown in Figure 5a. Otherwise, redox-labeled (such as ferrocene and methylene blue) DAb can be directly detected to quantify the antigen–CAb immunoreaction without adding external mediators or substrates in the electrolytes [46]. The detecting procedures are more straightforward and faster than the enzyme-labeled DAb.

The other labeled-based strategies are to use magnetic beads (MBs), metal NPs, GR, CNTs, or dendrimers as carriers for the simultaneous immobilization of DAb and electroactive species (such as enzymes, redox species, and soluble metals). After conjugating the antigen via the DAb, the carrier complex presents a synergistic effect to amplify the electrochemical signals, as shown in Figure 5b. Amperometry, voltammetry, or EIS are used to quantify the sandwich immunoreaction. Sadassivam et al. [47] adopted MBs as a carrier for immobilizing anti-carbohydrate antigen (CA125) CAb and HRP. After immunoreacting CA125 with the CAb-HRP@MBs, the collected complex of CA125/CAb-HRP@MBs was reacted with the aptasensors. Amperometry, CV, and EIS were used to quantify the CA125 concentration. Furthermore, electroactive metal NPs (Ag, Cu, Ce, and Pt) can be used as catalyzers to amplify the immunoreaction signal selectively, as shown in the left part of Figure 5b. Chen et al. [48] synthesized Ag@CeO_2_-Au nanocomposites as a carrier for DAb co-immobilization to perform sandwich immunoreaction on an anti-CEA-modified immunosensor. The cerium (III) and silver (I) autocatalytic reactions supplied a potential-selective signal to quantify the DAb-CEA-CAb immunoreaction.

**Figure 6:**
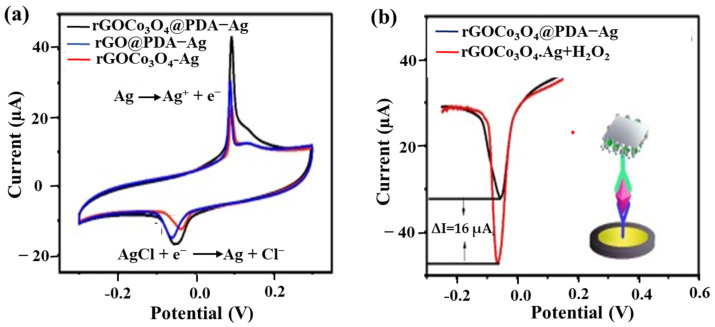
An example of using soluble AgNPs-based nanocomposite as a label for CEA detection. (**a**) CVs of RGO/Co_3_O_4_-Ag, RGO@PDA-Ag, and RGO/Co_3_O_4_@PDA-Ag in 0.1 M PBS (pH = 7.0) containing 0.1 M KCl. (**b**) DPV of RGO/Co_3_O_4_@PDA-Ag in 0.1 M pH 7.0 PBS containing 0.1 M KCl with (red line) or without (black line) 3 mM H_2_O_2_. Redrawn from [49].© Elsevier publishing.

Using soluble NP labels is a fascinating strategy with the native redox signal of soluble NPs as a probe for immunoreaction quantification, as shown in the right part of Figure 5b. For example, Liao et al. [49] synthesized RGO/Co_3_O_4_-Ag@PDA for immobilizing anti-CEA DAb, which immunoreacted with the CAb/AuNP/GCEs. The AgNPs are oxidized to Ag^+^ in positive potential scanning, and then Ag^+^ and Cl^−^ are combined to form an insoluble AgCl salt to adhere to the electrode surface. Subsequently, AgCl is reduced to AgNPs and Cl^−^ in the reverse potential scan, as shown in Figure 6a. The redox behavior of AgNPs immobilized on the nanocarrier can be a signal to probe the magnitude of sandwich immunoreaction.

Moreover, H_2_O_2_ addition can promote AgNP oxidation to obtain an increased reduction peak current, as shown in Figure 6b. The label-based nanocomposite types and their applications in electrochemical immunosensors are comprehensively discussed in Section 4, Nanomaterials Used as Labels.

## 3. Nanomaterials for Electrode Modification

Many nanomaterials, such as AuNPs and nanostructures (NSs), CNTs, GR, dendrimers, and their nanocomposites, have been applied in immunosensor fabrication. The conductive metal NPs, NSs, and CNTs can effectively increase the conductivity and roughness of the electrode surface. Dendrimers can form a 3D structure and have massive functionalities for antibody immobilization, promoting the number of bound antibodies [50]. Nanomaterial-modified electrodes are beneficial for developing label-free and label-based immunosensors. This section focuses on the fabrication and sensing properties of AuNPs/AuNSs, CNTs, and GR-modified immunosensors. 

### 3.1. Using AuNPs/AuNSs

AuNPs have been extensively used in electroanalytical chemistry and immunoassay because of their high surface-to-volume ratio, fast electron-transfer capabilities, and good biocompatibility. The surface of bare AuNPs is feasible for direct antibody adsorption. For example, Zhao et al. [51] used chemical vapor deposition to deposit GR on a monolithic 3D Ni foam and then directly deposited AuNPs on the GR/Ni foam via electroless deposition in an HAuCl_4_ solution through Au^3+^ reduction and Ni oxidation. The AuNPs/GR/Ni foam can adsorb antibodies as an electrochemical immunosensor. Yun et al. [52] also used electroless plating to form an AuNS layer on a pattered gold thin-film electrode for immobilizing protein A, which can adsorb the Fc portion of CAb. After performing sandwich immunoreaction with HRP-conjugated DAb, the immunosensors presented an LOD of 0.63 ng/mL in prohibitin 2 (PHB2)-spiked white blood cell lysates.

Furthermore, electrodeposition is an effective method for placing AuNPs and AuNSs on a conductive substrate. Beitollahi et al. [42] deposited AuNPs on an ionic liquid and graphite-mixed electrode for anti-prolactin CAb immobilization. Anti-prolactin DAb labeled with HRP was used to form sandwich immunoreaction for amperometric detection of human prolactin. The AuNPs were used to promote conductivity and surface area of electrodes for the thiolated linker binding, which can covalently bind CAb. In our previous works [38,53], the AuNS was formed on pre-oxidized screen-printed carbon electrodes (SPCEs) to increase the SPCEs roughness via two-step electrodeposition. The high rough AuNS/SPCEs-based immunosensors presented a greater sensitivity and a much lower LOD (4 fg/mL) than that (3 pg/mL) obtained at a planar Au disk electrode-based salbutamol immunosensor. The results indicate that AuNS deposition effectively increases the electrode roughness to lower the LOD significantly. Another strategy for immobilizing AuNPs on electrode surfaces is to adopt Au–S covalent bond and electrostatic adsorption. Specific thiolated groups (–SH) and positively charged groups of self-assembled monolayers (SAMs), polymers, and dendrimers are used for AuNP immobilization [54]. It is worth noting that SAM modification presents a simple way to immobilize AuNPs. Table 1 lists the sensing results of AuNPs or AuNS-based immunosensors, which showed ultralow LOD for different targets.

To further increase the number of AuNPs bound on the electrode surface, layer-by-layer assembled dendrimers are effective strategies for AuNP immobilization [55,56]. Amine-terminated polyamidoamine dendrimers have many functional groups at the periphery, encapsulating AuNPs in the dendrimers to the electrode surface [55]. Losada et al. [56] prepared AuNPs on ferrocenyl-dendrimer film on a GCE, which can directly oxidize NO_2_^−^ to NO_2_ for electrochemical determination of nitrite. We expect the mediator-AuNPs-dendrimer to be used as a nanocarrier for DAb immobilization to enlarge the antigen-CAb binding signal without adding an external mediator after sandwich immunoreaction.

Furthermore, electrodes modified with 3D nanostructures can provide an ultrahigh rough surface for biomolecule immobilization. Several methods of arranging 3D nanostructures on electrode surfaces have been applied to immunosensors [57]. Li et al. [58] constructed cucurbituril derivative-mediated 3D AuNPs structures for developing an impedimetric sensor for the label-free detection of 3-phenylpropylamine. Furthermore, AuNP formation can also accompany other metal reductions to form alloy nanoparticles. Li et al. [59] adopted 60Co gamma irradiation to prepare RGO-PtAu nanocomposite suspension. Then the Pt-AuNPs/RGO/GCEs were fabricated as immunosensors for label-free CEA detection via SWV. 

**Table 1:** Deposition methods of AuNPs or AuNSs for immunosensor fabrication.

Preparation Methods	Electrodes	Sensing Strategies/Techniques	LOD	LR	Refs.
Electrodeposition	Anti-prolactin/AuNPs/carbon paste electrode	Sandwich reaction with HRP-DAb and prolactin/DPV	12.5 mIU/L	25.0–2000.0 mIU/L	[42]
Electrodeposition	Anti-NSE/AuNPs-MoS_2_-rGO/GCE ^1^	Sandwich reaction with DAb/ CoFe_2_O_4_-Ag, and NSE by SWV	3 fg/mL	0.01–1.00 pg/mL	[60]
Electrodeposition	Anti-HSA/AuNPs/PpPD/PEDOT-PSS-Fc /SPCEs ^2^	Label-free/DPV	0.54 fg/mL	1–10 ng/mL	[61]
Electroless plating	Anti-PHB2/PA/AuNS/AuE	Sandwich reaction with HRP-Dab and PHB2/SWV	40 pg/mL	0–10 ng/mL	[52]
Electrostatic adsorption	Anti-CA153/PPy-AuNPs-luminol/ITO ^3^	Label-free/EIS & electrochemiluminescence	5.8 × 10^−4^ U/mL	0.001–700 U/mL	[62]
Multi-layer electrostatic adsorption	Anti-PSA-GSH-AuNPs/PEI/PVS/PEI/MUA/AuE ^4^	Label-free/EIS	0.17 ng/mL	0.1–20 ng/mL	[63]

^1^ Anti-NSE: anti-neuron-specific enolase antibody; ^2^ Anti-HSA/AuNPs/PpPD/PEDOT-PSS-Fc: anti-human serum albumin/AuNPs/poly(para-phenylenediamine)/poly(3,4-ethylenedioxythiophene)-poly(styrene sulfonate)-ferrocene nanocomposite; ^3^ Anti-CA153/PPy-AuNPs-luminol: anti-carbohydrate antigen 153/polypyrrole-luminol-AuNPs; ^4^ Anti-PSA-GSH-AuNPs/PEI/PVS/PEI/MUA: anti-prostate specific antigen-glutathione-AuNPs/poly(ethyleneimine)/poly(vinylsulfonicacid)/poly(ethyleneimine)/mercaptoundecanoic acid.

### 3.2. Using CNTs

CNTs have interesting chemical and physical properties, including high electrical conductance, large surface area, good biocompatibility, and functionalization potential. They have garnered considerable attention in electrochemical immunosensors [64,65]. CNTs have considerable molecular weight, conductivity, and high surface area, which allows CNTs to be placed alone, mixed with polymers, deposited with conductive materials (metal or GR), or electropolymerized with monomers for the surface deposition of electrodes. The CNT-hybrid nanocomposites can directly attach to electrode surfaces due to van der Waals force attraction. GO, RGO, or metal nanoparticles are commonly mixed with CNTs to produce a synergistic effect to enhance the electrochemical response [66].

In addition, CNT composites with Teflon, poly(l-arginine), Nafion, or chitosan (CS) can provide a simple and flexible method for fabricating immunosensors [67,68]. The CNT ink can be directly dripped on an electrode to produce a highly conductive and rough surface of the electrodes for antibody immobilization. For example, Sun et al. [69] chemically deposited AuNPs on polyethyleneimine (PEI)-coated MWCNTs for protein A immobilization. The Fc fragment of CAb can directionally adsorb anti-kidney bean lectin (KBL) on the AuNPs-PEI-MWCNT/GCEs to increase the binding efficiency of KBL. Deiminiat et al. [70] synthesized carboxylic acid-functionalized MWCNTs(COOH-MWCNTs) to form COOH-MWCNTs-AuNPs nanocomposite for anti-bisphenol A (BPA) aptamer modification, which can perform label-free detection of BPA in mineral water, orange juice, and milk. The metal NPs decorated on CNTs can increase the binding sites of biorecognition molecules and promote redox response. Shahrokhian et al. [71] developed a sensitive voltammetry sensor for determining isoxsuprine based on the MWCNT/AgNPs-modified GCEs. The anodic peak current response of AgNPs measured by LSV is more sensitive than the native oxidative current of isoxsuprine. Xing et al. [72] electrodeposited Prussian blue on COOH-MWCNTs-coated GCE. The Prussian blue metal-organic framework (MOF)/COOH-MWCNTs/GCEs presented excellent stability, reproducibility, and recovery for the CV-based detection of bisphenol B in actual river samples. Ultrasonication is an effective method of preparing CNT-based nanocomposites for electrode modification [73,74].

**Figure 7:**
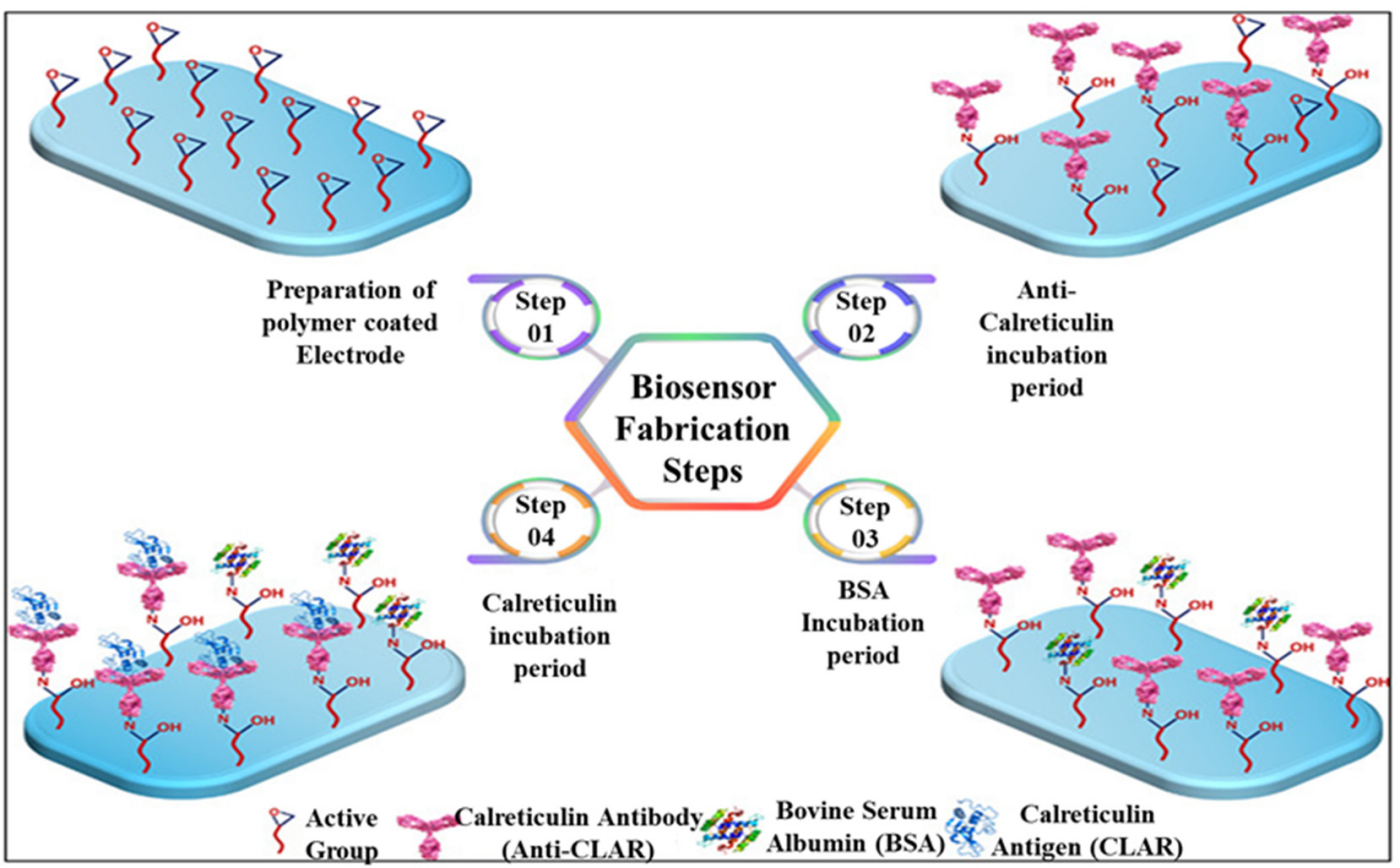
Schematic of the label-free immunosensor for CALR construction process with disposable electrode coated with SWCNTs-PPepx nanocomposite. Redrawn from [75]. © ACS Publishing.

Furthermore, electropolymerization can locally deposit CNTs on the surface of the working electrode. Mixing electroactive monomers, such as pyrrole [75,76] and aniline [77], with CNTs is essential for electropolymerization. Aydın et al. [75] mixed SWCNT and oxiran-2-yl methyl 3-(1H-pyrrol-1-yl) propanoate monomer (Pepx) prepared from 1- pyrrolepropionitrile via the two reaction steps of hydrolysis and esterification and electrodeposited the conductive SWCNTs-PPepx nanocomposite on an ITO electrode for antibody immobilization. The preparation procedures are shown in Figure 7. The EIS-based calreticulin immunosensor presented excellent linear ranges of 0.015–60 pg/mL and an ultralow LOD of 4.6 fg/mL. The different fabrication methods of CNT-based immunosensors and their sensing performance are compared in Table 2. In summary, CNTs can be used as an excellent conductive substrate and mixed with GR, metal NPs, MOFs, charged polymers, or redox polymers to form hybrid nanocomposites to present synergistic reactions for promoting the binding number of antibodies and facilitating electrochemical detection, which show greatly promising potential in sensing applications.

### 3.3. Using GR/RGO

Immunosensors constructed by GR-based nanocomposites have attracted attention due to 2D structures, fast electron transportation, large surface area, and good biocompatibility. However, some inherent disadvantages of GR need to be overcome, such as hydrophobicity and easy aggregation in an aqueous solution. Several surface modification strategies, such as metal nanoparticle deposition, strong acid oxidation, and polymer mixture, are used to improve the hydrophilicity and increase the biocompatible binding sites of GR-based composites [79,80]. Sun et al. [81] used chemical vapor deposition to grow vertical GR on a GCE and electrodeposited AuPtNPs on the vertical GR nanosheets as binding sites of CAb for label-free detection of alpha-fetoprotein (AFP). Low et al. [82] used a solvothermal method to prepare GR/zinc oxide nanocomposites as the interface of an electrochemical genosensor to detect a single standard RNA. Salimi et al. [83] synthesized amine-functionalized GR using a hydrothermal method in NH_3_ and sodium bisulfite. An RNA probe could be immobilized on amine-GR/GCE for label-free EIS-based detection of miRNA-155. 

Compared to GR, GO has superior hydrophilicity and more accessible surface functionality for modifying biorecognition molecules due to its abundant oxygen-containing functional groups, such as hydroxyl and epoxy groups on its basal plane and a carboxyl group at its edge. GO can be prepared from graphite powder via the strong oxidation of HNO_3_, KMnO_4_, and H_2_SO_4_ according to the method reported by Hummers and Offerman [84]. Subsequently, the COOH-GR slurry was drop-casted on SPCEs for the electrodeposition of 2-aminobenzylamine, and then 1-ethyl-3-(3-dimethylminoprpyl) carbodiimide (EDC)/N-hydroxysuccinimide (NHS)-activated CAb was immobilized on the 2-aminobenzylamine/COOH-GR/SPCEs. EIS was used to quantify the direct immunoreaction of parathion. Furthermore, the bottom-up syntheses can also produce GO, such as chemical vapor deposition and epitaxial growth on silicon carbide wafers [85]. The high hydrophilicity of GO makes it easy to suspend in an aqueous solution for preparing GO-based nanocomposites. Yuvashree et al. [86] mixed GO and CS to modify a GCE to produce an H_2_O_2_ sensor. GO has also been used as a substrate for gold nanocrystal deposition to exhibit synergistic behavior for the electrocatalytic reaction of dopamine, uric acid, and 4-aminophenol [87]. Pal and Khan electrodeposited AuNPs on a GO-coating Pt electrode to enhance the conductivity. EDC–NHS mixture was used to activate the GO surface for immobilizing anti-PSA CAb. DPV was performed for the label-free detection of PSA [88].

**Figure 8:**
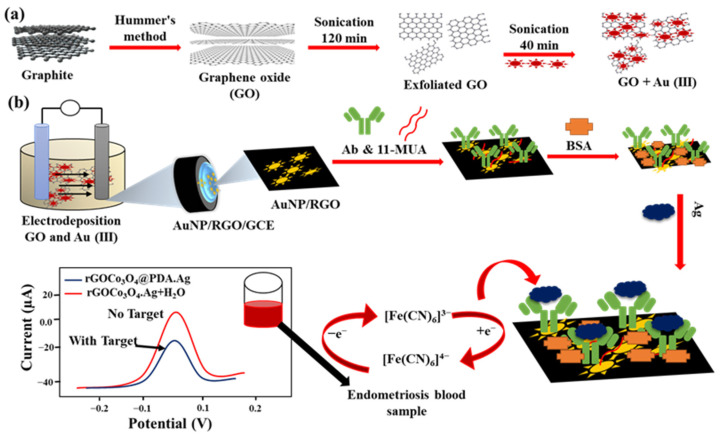
Showing the preparation and exfoliation of GO+Au^3+^ in (**a**) and the fabrication of AuNPs/RGO/Ab/BSA/Ag/GCE and CA125 detection for endometriosis in (**b**). Redrawn from [93]. © ACS Publishing.

However, the weaker conductivity of GO due to higher sp^3^ carbon and abundant oxygen groups hinders its application in constructing an electrochemical immunosensor [89]. Therefore, the procedures to transfer GO to RGO become vital for electrochemical biosensors. Currently, many studies are developing controllable strategies, including chemical reduction and electro-reduction, to fabricate RGO-based immunosensors [90,91]. Jozghorbani et al. [92] chemically reduced GO in an ascorbic acid and ammonium hydroxide mixture and dripped the RGO onto GCE for anti-CEA immobilization. The high conductivity and roughness of RGO/GCE are beneficial for the label-free detection of CEA immuno-reaction through CV and EIS. Figure 8 represents a single-step assembly of an AuNPs/RGO-based SWV-detected immunoassay for detecting an endometriosis biomarker in clinical diagnosis [93].

**Figure 9:**
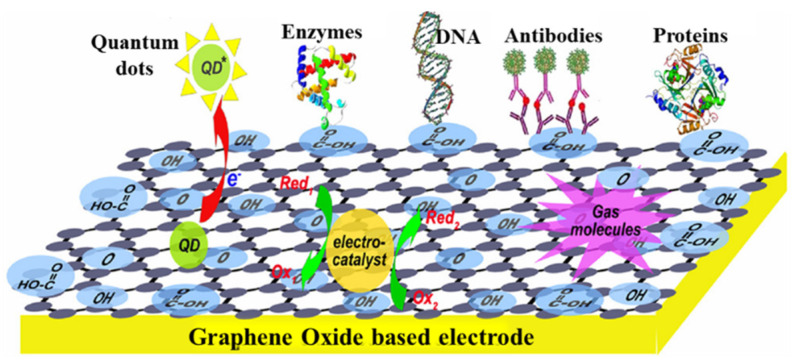
The scheme shows GO-based electrodes for electrochemical applications. Redrawn from [95]. © ACS Publishing.

In addition to the GR, GO, and RGO, exfoliated graphite nanoplatelets, composed of stacked 2D graphene sheets, have high conductivity and roughness suitable as a surface modifier of an electrode due to the SP^2^ hybridized carbons. Zanato et al. [94] deposited AgNPs-Nafion on the surface of exfoliated graphite nanoplatelets and adsorbed the anti-microcystin-LR antibody to form a nanocomposite. Subsequently, the nanocomposite was dripped onto a cleaned GCE as an immunosensor for label-free detection of microcystin-LR by using SWV and EIS measurement. The AgNPs can be directly oxidized as a probe to indicate antigen–antibody conjugation. Exfoliated graphite nanoplatelets supply a large surface for more AgNP adsorption, drastically increasing the background signal. The peak current of SWV decreased with increasing microcystin-LR concentration with LR of 0.5–500 ng/mL and LOD of 0.017 ng/mL. As mentioned in Nanda et al. [95], GO-based electrodes have been shown to have wide applications due to the advantages of good flexibility for soft electronics, different oxygen-containing functionalities for biomolecule immobilization, and excellent conductivity, as shown in Figure 9.

## 4. Nanomaterials Used as Labels

In sandwich immunoreaction, enzymes or redox species can be directly conjugated with DAb as a probe to report the antigen–CAb interaction. Due to the size, functionality, and bioactivity of DAb, the bound number of the electroactive probe or catalyzer has a practical limit. Furthermore, the chemicals used for the probe conjugation may damage the DAb immunoactivity. In contrast, nanomaterial carriers can supply a large surface for immobilizing DAb and different electroactive species to amplify the antigen–CAb immunoreaction signal. The nanomaterial carriers can provide a biocompatible surface for DAb and a high-conductive interface for electrochemical detection. According to the functions, the probe species conjugated on the nanomaterial carriers can be divided into three categories: (1) enzyme [47,96,97] or electroactive catalyzer [98,99,100,101] with the addition of reactants in the electrolyte; (2) electroactive species with direct redox reaction [48,102,103,104], (3) metal NPs oxidized to form soluble metal ions after strong-acid dissolution [105,106,107,108]. The nanomaterial carriers conjugated with different electroactive species and DAb present excellent specificity through sandwich or competitive immunoreactions and reduce the signal interference induced by non-specific adsorption on the final electrochemical results

### 4.1. Nanomaterial Carriers with Enzymatic/Electroactive Catalyzer

Nanomaterials simultaneously conjugated with enzymes and DAb are one of the most prevalent strategies to amplify the signal of antibody–antigen interactions through substrate catalysis to produce electroactive products. HRP [96], GOx [43,44], and lactate oxidase (LaOx) [97] frequently accompany DAb to be immobilized on nanomaterial carriers. Table 3 shows the different substrates added to the electrolyte and the related electroactive products. In these sandwich immunosensors, the product concentration is proportional to the number of immunoreacted antigens, the enzyme-conjugated complex, and the catalyzing time. Therefore, an intuitive current increase can be obtained by voltammetry and amperometry. Furthermore, metal NPs are used as an alternative for substrate catalysis, especially for H_2_O_2_ reduction. Trimetallic yolk-shell Au@AgPt nanocubes [98], Fe_3_O_4_/Au@AgNPs [99], Au@SiO_2_/Cu_2_O nanoparticles [100], and Au@Ag-Cu_2_O [101] were used to enhance H_2_O_2_ reduction. High electrocatalytic metals, such as Pt, Ag, and Cu_2_O, play an essential role in H_2_O_2_ electroreduction. The other nanomaterials of the nanocomposites present a synergistic effect on electrocatalysis due to their high conductivity and electron density. In contrast, the electrocatalytic metal-based sandwich immunosensors exhibit superior sensing properties compared to the enzyme-conjugated sandwich immunosensors with lower LOD and wider LR, as shown in Table 3. The phenomenon is attributed to the better catalytic efficiency of the electrocatalytic metal NPs.

### 4.2. Nanomaterial Carriers with Electroactive Species for Direct Redox Reaction

After sandwich immunoreaction, direct electron transfer of redox species from the DAb-conjugated nanomaterial carriers to the electrodes is a more straightforward detecting method than enzymatic/electroactive catalyzer-based detection. Redox species, such as methylene blue [28], Thi [102], ferrocene [33], toluidine blue (TB) [103], AgNPs [48,49], and Cu-based NPs [104], are co-immobilized on nanomaterials with DAb to supply an electrochemical signal. The voltametric peak current significantly increases when the DAb-labeled nanomaterial carriers react with the antigen conjugated on the CAb-immobilized electrodes. However, the non-specific adsorption of nanomaterial carriers may cause a false positive signal due to the large contact area and molecular size. Table 3 compares the sensing properties of immunosensors after sandwich immunoreaction with the different kinds of redox species-conjugated nanomaterial carriers. Among the redox species, AgNPs present excellent oxidative signals through direct electrooxidation of Ag-Ag^+^ [48] or H_2_O_2_-enhanced oxidation [49], which can reach sub-Pico molar level LOD. Liao et al. [49] developed a dual catalytic signal enhancer based on external H_2_O_2_ and AgNPs co-immobilized on the anti-CEA DAb, Co_3_O_4_, and polydopamine (PDA)-modified RGO. The LOD of the CEA immunosensors was as low as 0.17 pg/mL. The results show that AgNPs can be a promising electroactive probe to report immunoreaction intensity directly. Krishnan et al. [109] fabricated HRP/Thi dual-labeled mesoporous silica nanospheres conjugated with Au nanorod and DAb, which immunoreacted with CEA and the CAb/NiO@Au/GR-modified ITO electrode. The dual signal amplification permitted CEA detection with 5.25 fg/mL LOD using DPV detection.

### 4.3. Nanomaterial Carriers with Soluble NPs

The semiconductor NPs conjugated on nanomaterial carriers, such as CdS [105,108] and CdSe [106,107], can be dissolved by strong acids, such as HCl, and HNO_3_, to release Cd^2+^ ions. Then ASV is used to detect sensitively the reduction signal of metal ions. Tocco et al. [105] conjugated CdS nanocrystals on phage capsid to detect molinate herbicide in river water using CAb/polynitroaniline-modified GCE. After performing sandwich immunoreaction, a 0.1 M HCl solution was added to obtain Cd^2+^, which can be detected by square wave ASV. Qin et al. [108] prepared the CAb/β-cyclodextrin-graphene sheets (CD-GS) nanocomposite/GCEs and the DAb-ZnO-MWCNTs nanocarrier, respectively. After performing the sandwich immunoreaction with the antigen of human heart-type fatty-acid-binding protein (FABP), Cd(NO_3_)_2_ and thioacetamide were used to deposit CdS on the ZnO surface chemically, and then HNO_3_ was used to produce Cd^2+^ as a signal reporter. In these studies, the soluble NPs can be initially immobilized on the nanocarrier for sandwich immunoreaction or subsequently deposited on the nanocarrier immunoreacted with antigens and immunosensors. This chemically depositing CdS technique is feasible to control the number of CdS NPs and suitable for different nanocarriers, which shows promising potential for ultrasensitive detection of analytes. The immunosensors adopted with different soluble metal NPs are compared in Table 3. 

**Table 3:** Deposition methods by labeled nanomaterials for immunosensor fabrication.

Label-Based Nanomaterials	Analyte/Electrodes	Sensing Strategies/Techniques	LOD	LR	Refs.
Anti-HFA ^1^ DAb & HRP/COOH-MWCNT	HFA/CAb-biotin/streptavidin/SPCE	H_2_O_2_/HRP/hydroquinone/amperometry	16 pg/mL	20–2000 pg/mL	[96]
Anti-CA125 DAb/AuNP-LaOx ^2^	CA125/CAb/CS-AuNP/MWCNT-GO/GCE	H_2_O_2_/LaOx/lactic acid/amperometry	2 mU/mL	0.01–100 U/mL	[97]
Anti-CEA DAb/MoS_2_ NFs/Au@AgPt YNCs ^3^	CEA/CAb/MoS_2_/Au@AgPt YNCs/GCE	Enhanced H_2_O_2_ reduction via AgPt/amperometry	3.09 fg/mL	1 × 10^5^ –100 ng/mL	[98]
Anti-CEA DAb/GR sheet-Fe_3_O_4_/Au@Ag/Ni^2+^	CEA/CAb/AuNPs/GCE	Enhanced H_2_O_2_ reduction via Ni^2+^/amperometry	69.7 fg/mL	1 × 10^−4^–100 ng/mL	[99]
Anti-CEA DAb/Au@SiO_2_/Cu_2_O	CEA/CAb/Ag/g-C_3_N_4_ ^4^ /GCE	Enhanced H_2_O_2_ reduction via Cu_2_O/amperometry	3.8 fg/mL	1 × 10^−5^−80 ng/mL	[100]
Anti-PSA DAb/Au@Ag-Cu_2_O	PSA/CAb/Au@N-GQDs/GCE	Enhanced H_2_O_2_ reduction via Cu_2_O/amperometry	3 fg/mL	1 × 10^−5^−100 ng/mL	[101]
Anti-cTnI DAb/N,S-cGO/L-lys/AuNR@Pt MBs/Thi ^5^	cTnI/CAb/AuNR@PDA/GCE	Direct reduction of Thi/ amperometry and DPV	16.7 fg/mL	5 × 10^−5^–250 ng/mL	[102]
Anti-CEA DAb/Ag@CeO_2_ core-shell-Au NPs	CEA/CAb/AuNPs/GCE	Ag-CeO_2_ direct redox/CV & EIS	32 fg/mL	1 × 10^−4^–5 ng/mL	[48]
Anti-CA125 DAb-TB/Suc-CS@MNP ^6^	CA125/CAb/PAMAM ^7^ /AuNP-3D RGO-MWCNT	Direct reduction of TB/SWV	6 μU/mL	0.0005–75 U/mL	[103]
Carbaryl hapten@CuNP-CS	Carbaryl/CAb/AuNP/GCE	Direct oxidation of CuNPs after immuno-competition/linear sweep ASV	0.05 ng/mL	0.5–20.0 ng/mL	[104]
Anti-CEA DAb/RGO/Co_3_O_4_-Ag@ PDA	CEA/CAb/AuNP/GCEs	Ag-Ag+ redox with H_2_O_2_ enhancement/DPV	0.17 pg/mL	0.0005–80 ng/mL	[49]
CdS nanocrystals/phage	Molinate/14D7 CAb/polynitroaniline/GCE	CdS-Cd^2+^ with HCl dissolution/square wave ASV	34 pg/mL	0.1–10 ng/mL	[105]
Anti-casein biotin-CAb/Streptavidin/CdSe/ZnS QDs	Bovine casein/Bovine casein/Sb_2_O_5_-SnO_2_/SPCEs	CdSe-Cd^2+^ after immuno-competition with HCl dissolution/ASV	0.07 % (*v*/*v*)	0.1–10% (*v*/*v*) Cow’s milk in ewe/goat’s cheese	[106]
anti-HE4 CAb/CdSe/ZnS QDs	HE4 ^8^ /DAb/MBs/Hg/SPCEs	CdSe-Cd^2+^ after immuno-competition with HCl dissolution/ASV	2 pM	20–40 nM	[107]
Anit-FABP DAb/CdS- ZnO-MWCNTs	FABP/CAb/CD-GS/GCE ^9^	CdS-Cd^2+^ with HNO_3_ dissolution/ASV	0.3 fg/mL	1.3–130 ng/mL	[108]

^1^ HFA: human fetuin A; ^2^ LaOx: lactate oxidase; ^3^ MoS_2_ NFs/Au@AgPt YNCs: MoS_2_ nanoflowers/trimetallic yolk-shell Au@AgPt nanocubes; ^4^ g-C_3_N_4_: graphitic carbon nitride; ^5^ Anti-cTnI/cGO/L-lys/Au@Pt MBs/Thi: anti-cardiac troponin I/nitrogen/sulfur co-doped graphene oxide/L-lysine/Au nanorod@Pt core-shell multi-branched nanoparticles/thionine; ^6^ TB/Suc-CS@MB; toluidine blue */O*-succinyl-chitosan-magnetic nanoparticles; ^7^ PAMAM: polyamidoamine; ^8^ HE4: human epididymis protein 4; ^9^ FABP/CD-GS/: human heart-type fatty-acid-binding protein/β-cyclodextrin-graphene sheets.

## 5. Other Trends and Challenges in Electrochemical Immunosensors

In recent years, new electrochemical immunosensors have not only focused on nanomaterials used in the fabrication of electrodes or labels but also on practical aspects, such as low-cost, disposability and mass produced electrodes, antifouling for non-specific adsorption, high surface nanocarriers, multi-targets detection in one sensing interface, and microfluidic integration for rapid or high-throughput detection. We present some previous studies as examples to elucidate the trends and challenges.

**Figure 10:**
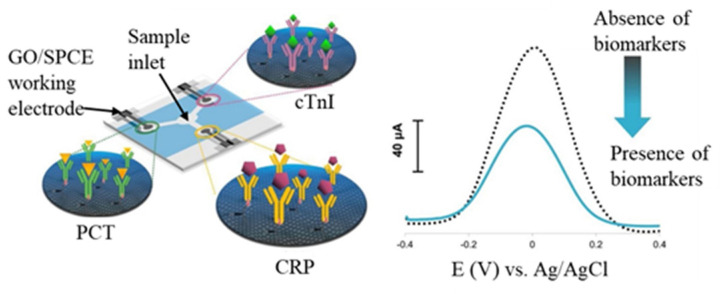
Scheme of multiplexed electrochemical paper-based analytical devices (ePADs) with three individual immunosensors. The label-free DPV peak current decreases after immunoreaction. Redrawn from [118]. ©Elsevier Publishing.

(1) Although GCE is durable and frequently used for immunosensor construction, SPCEs, possessing the benefits of low cost and ease of massive production, present great promise to develop disposable immunosensors. The nanocarrier can be dripped on the SPCEs as substrate for antibody immobilization. Wei et al. [110] synthesized RGO/Prussian blue/core-shell Au@PtNPs as a nanocarrier for drop-coating SPCEs. The anti-hepatitis B antibody could be directly adsorbed on the nanocarrier surface, and the Prussian blue served as an electron-transfer mediator. After immunoreaction with the hepatitis B surface antigen, the label-free LOD measured by DPV was 80 pg/mL. Furthermore, Malla et al. [111] fixed a magnet to the backside of SPCEs for adsorbing HRP-CAb-modified MBs conjugated with parathyroid hormone antigen and then performed the catalysis of H_2_O_2_ and hydroquinone with SWV detection to obtain an LOD of 11.56 pg/mL. The drip-coating fixation or the magnetic adsorption of CAb-modified nanocomposite carriers on SPCEs can simplify the preparation of SPCE-based disposable immunosensors. Although SPCEs are prevailing in the development of disposable point-of-care testing strips, the activation or the peroxidation procedures of the SPCE surface still take up much time before use. Oxygen plasma treatment is an alternative for mass production. Subsequently, a sealing package for long-term storage is essential after plasma treatment.
(2) Preventing the effect of non-specific adsorption on label-free electrochemical immunosensors from versatile molecules of actual samples is an essential issue. Antifouling materials, such as poly(ethylene glycol) and zwitterionic polymers [112], block the electrode surface to reduce non-specific adsorption. Wang and Hui electrodeposited polyaniline nanowires on a GCE to produce a highly rough surface, and photopolymerized zwitterionic poly(carboxybetaine methacrylate) (polyCBMA) on the polyaniline nanowire to obtain a hierarchical structure. After chemical activation, the anti-CEA antibody was covalently immobilized on polyCBMA without extra surface blocking. The DPV-based immunosensors presented an ultralow LOD of 3.05 fg/mL and an impressive antifouling ability from cow’s milk, saliva, bovine fetal serum, and human serum [113]. The modification technique of antibody and polyCBMA supplies promising potential for the antifouling treatment of immunosensors.
(3) Multiplexed detection in clinical diagnosis, agricultural pesticide/herbicide residue, and environmental toxins has considerable importance due to their excellent analytical efficiency compared with parallel single-analyte assays. Two kinds of multiplexed detecting strategies have been developed. One is to use different multi-detectors placed on the same substrate. Serafín et al. [114] separated immobilized anti-tau protein (tau) CAb and anti-TAR DNA-binding protein 43 (TDP-43) CAb on the two 3D-Au-PAMAM-modified working electrodes of the SPCEs. After sandwich immunoreaction, the HRP-conjugated DAb can quantify the tau and TDP-43 in raw plasma samples by catalyzing the H_2_O_2_/hydroquinone reaction with amperometric detection. Furthermore, Salahandish et al. [115] developed dual-immunosensors for the label-free detection of SARS-CoV-2 nucleocapsid protein by EIS. The other multiplexed technique uses biorecognition molecules, tagging different electroactive mediators on the identical sensing interface. Shen et al. [116] tagged anthraquinone on the VEGF-aptamer, methyl blue on the IFN-γ aptamer, and ferrocene on the TNF-α-aptamer to achieve multiplex detection, respectively. The three kinds of aptamers were biotinylated to immobilize them on the single streptavidin/GO/AuE. Then, SWV was performed to obtain the redox signal of anthraquinone, methyl blue, and ferrocene at −0.45 V, −0.26 V, and 0.25 V before and after the label-free immunoreaction. Compared to the single electrode immobilized by multi-CAbs, the multi-electrodes with different CAb immobilization are easier to control the density of CAbs to obtain better sensing properties.
(4) The immunosensors integrating fluidic transportation can promote immunoreaction efficiency in shorter immunoreaction time and reduce detecting procedures. Lin et al. [41] fabricated an impedimetric affinity sensing chip integrated with an AC electrokinetic flow vortex. The protein A-antibody affinity time can reach the plateau in 8 min with AC electrokinetic flow. The corresponding EIS-R_et_ value of the affinity plateau was 2.26 times larger than that obtained in an unstirred solution. Furthermore, the paper-based immunoassay becomes an exciting alternative for constructing disposable, low-cost, and eco-friendly analytical devices due to flexibility, lightness, capillary-driven flow, and affordability in an austere environment. Shu et al. [117] utilized a paper-based electrochemical immunosensing device for the label-free detection of AFP. The Ni-Co MOF nanosheets were modified with CNT and streptavidin and then coated onto a GR-printed working electrode for biotinylated CAb immobilization. After immunoreaction with samples conducted through vertical flow, the H_2_O_2_/hydroquinone mixture was dripped to produce a DPV signal via Ni-Co MOF catalysis. Furthermore, Boonkaew et al. [118] constructed triple three-electrode SPCEs in triple channels in an identical substrate to form multiplexed electrochemical paper-based analytical devices (ePADs), as shown in Figure 10. Three kinds of antibodies were respectively immobilized on the different GO/SPCEs to capture C-reactive protein (CRP), cardiac troponin I (cTnI), and procalcitonin (PCT) of the cardiovascular disease biomarkers. After immunoreaction, the redox solution was dripped into the central inlet and conducted to the sensing region via lateral flow for DPV detection. The multiplexed ePADs can detect C-reactive protein, cTnI, and PCT with corresponding LODs of 0.38 ng/mL, 0.16 pg/mL, and 0.27 pg/mL, respectively. The design and fabrication of ePADs have promising potential in constructing a multiplexed point-of-care testing device.

## 6. Conclusions

This work reviews the articles related to the sensing strategies of electrochemical immunosensors using nanomaterials. AuNPs, AuNS, CNTs, and GR-based nanomaterials can be used in label-free immunosensors as the CAb-immobilization substrate to supply a highly conductive and rough surface or a mediator-embedded interface. Potentiometry, voltammetry, and EIS are suitable for label-free immunosensors. Furthermore, nanomaterials can be used as a carrier for the immobilization of DAb, catalyzers, redox mediators, or soluble metal NPs, which act as a nanocomposite label in a sandwich or competitive immunoreaction to amplify the signal of antigen–CAb conjugation. Amperometry and voltammetry are frequently used to quantify the immunoreaction results. It is worth noting that the combination of the soluble metal NP-modified nanocarrier and ASV detection can produce the best sensitivity and lowest LOD. 

Emerging techniques, including the cost-effective production of electrodes, antifouling treatment, multiplexed detection, disposable and environment-friendly paper-based substrates, microfluidic integration, and miniature potentiostat chips, drive electrochemical immunosensors to present versatile applications in practical fields. Moreover, the nanomaterial characteristics of high roughness, good biocompatibility, electrocatalysis, and excellent conductivity, can promote the emerging electrochemical immunosensors with more distinguished sensing properties. 

## Figures and Tables

**Table 2:** Preparation of CNT-based nanocomposites for immunosensor fabrication and their sensing properties.

CNT Hybrid Preparation Methods	Electrodes	Sensing Strategies	LOD	LR	Refs.
Chemical reduction of AuNPs on MWCNT	Anti-KBL/PA/AuNPs-PEI-MWCNTs/GCE	Label-free/DPV	23 ng/mL	0.05−100 μg/mL	[69]
Chemically synthesized COOH-MWCNTs/AuNPs	Anti-BPA aptamer/COOH-MWCNTs/AuNPs/AuE	Label-free SWV	114 pg/mL	22.8–2283 pg/mL	[70]
Ultrasonication	α-fetoprotein/N-GQD ^1^ @SWCNTs/Anti-AFP/BSA/GCE	Label-free/CV and EIS	0.25 pg/mL	0.001–200 ng/mL	[73]
CNTs/PEI/GE ^2^ via LBL fashion	CA19-9/PEI-CNTs/EDC-NHS	Label-free/EIS	0.35 U/mL	0.05–0.5 U/mL	[78]
RGO/CNF and RGO/CNT via sonication and hydrothermal reaction	RGO-CNT-Thi/anti-CA125/AuNPs/GCE	Sandwich reaction/DPV	0.28 pg/mL	1–3.2 ng/mL	[74]
Electropolymerization	Anti-calreticulin/SWCNTs-PPepx /ITO	Label-free/EIS	4.6 fg/mL	0.015–60 pg/mL	[75]
Electropolymerization	Anti-CysC/PPy-CNTs/ IDE ^3^	Label-free/Capacitance	28 ng/mL	30–300 ng/mL	[76]

^1^ N-GQD: nitrogen doped graphene quantum dot; ^2^ CNTs/PEI/GE: CNTs/polyethyleneimine/Gold electrode; ^3^ Anti-CysC/PPy/CNTs/IDE: anti-cystatin-C/polypyrrole/CNTs/interdigitated electrode.

## Data Availability

Not applicable.
